# GSK3 Kinase Inhibitor, CHIR, Suppress Transcription of Tissue Specific POU2F1 Isoform in Burkitt Namalwa Lymphoma Cells

**DOI:** 10.1134/S1607672921010087

**Published:** 2021-03-10

**Authors:** E. V. Pankratova, T. N. Portseva, A. A. Makarova, Yu. V. Ilyin, A. G. Stepchenko, S. G. Georgieva

**Affiliations:** Engelhardt Institute of Molecular Biology, Russian Academy of Sciences, Moscow, Russia

**Keywords:** transcription factor POU2F1(Oct-1), alternative promoters, GSK3 kinase

## Abstract

POU2F1 (Oct-1) is a transcription factor, the overexpression of which is found in many human malignant tumors; a significant increase in its level in cells determines the malignant potential of the tumor. POU2F1 is represented in cells by several isoforms that are transcribed from alternative promoters. In Burkitt’s B-cell lymphoma Namalwa, the concentration of tissue-specific isoform Oct-1L is several times higher than in normal B cells. We tested the potential to inhibit the transcription of individual Oct-1 isoforms using the GSK3 kinase inhibitor CHIR, an aminopyrimidine derivative. We have shown that CHIR specifically affects the expression of the tissue-specific isoform Oct-1L, significantly reducing the level of mRNA and Oct-1L protein. However, CHIR does not change the amount of mRNA and protein of the ubiquitous isoform Oct-1A in Namalwa tumor cells. The results obtained show that it is possible to develop a system for selective inhibition of Oct-1 transcription factor isoforms in human cells to suppress drug resistance of tumor cells with a high POU2F1 content.

The POU2F1 (Oct-1) protein belongs to the POU-family of transcription factors [1, 2]. Oct-1 is expressed in all human cells and controls embryonic development, differentiation, and cellular response to stress [3, 4]. The ability of Oct-1 to regulate a wide range of functionally different genes and processes is based on its ability to interact with various DNA sites [5] and undergo multiple posttranslational modifications. Oct-1 isoforms differing in the sequence of the N-terminal domain are transcribed from alternative promoters and regulate a large number of target genes, both individual for each isoform and common for all Oct-1 isoforms [4, 6–8].

A high level of POU2F1 (Oct-1) ensures cell resistance to stress under oxidative and cytotoxic stresses, as well as at radiation exposure, and the degree of its involvement in these processes depends on its concentration in the cell [3, 9]. POU2F1 (Oct-1) is a pro-oncogenic factor and has prognostic value in the development and treatment of a wide range of malignant tumors. A high level of POU2F1 (Oct-1) expression in a tumor is associated with a poor diagnostic prognosis. Thus, Oct-1 is a significant transcription factor that determines the malignant potential of a tumor [9, 10].

An increased expression of Oct-1 is detected in a large number of tumor types; however, a rather high level of its expression is also observed in many types of non-malignant cells and tissues. This complicates the use of anticancer therapy based on complete or partial suppression of Oct-1 without taking into account the specific isoform that is suppressed.

Oct-1 isoforms are expressed in normal cells at certain ratios that are relatively stable for certain cell types [4, 7, 8]. Disturbance in the expression level of some Oct-1 isoforms is observed in many types of tumor tissues and is associated with dysregulation of alternative promoters of the *POU2F1* (*Oct-1*) gene in tumor cells. For example, in Burkitt Namalwa B-cell lymphoma, the concentration of the tissue-specific isoform Oct-1L is several times higher than in normal B cells [4, 8].

The existence of alternative promoters in the *POU2F1* (*Oct-1*) gene makes it promising to influence not only the expression of total Oct-1, but also the expression of its individual isoforms, the level of which increases in tumor cells.

In this work, we studied the possibility of selectively influencing the expression of Oct-1A and Oct-1L isoforms transcribed from alternative promoters. The analysis of the distribution of binding sites in the regions of two alternative promoters of the *POU2F1* (*Oct-1*) gene showed that the transcription of this gene may be under the control of the target proteins for GSK3 (glycogen synthase kinase-3), TCF/LEF, and FOXO. Both promoters contain binding sites for transcription factors of the TCF/LEF family, through which B-catenin is anchored on the promoters of the target genes of the Wnt signaling pathway, and the L promoter also contains the binding sites for FOXO. Based on the analysis of the distribution of binding sites, we assumed that substances that can influence the activity of GSK3 can selectively influence the activity of these promoters.

For the transcription factors POU2F1 and GSK3, their involvement in carcinogenesis has been shown. However, the relationship between GSK3 and the *POU2F1* gene in the regulation of carcinogenesis has not been studied. The functions of GSK3 in tumor development are ambiguous. GSK3 can be both a tumor suppressor and an activator of tumor growth, depending on the tumor type. A high level of GSK3 in the cell inhibits tumor growth in breast tumors and melanoma, but enhances the growth of pancreatic tumors and in leukemia.

The pleiotropic effect of GSK3 is associated with its central role in different signaling pathways (Notch, Wnt, Hedgehog, and NF-kB) [11].

We have tested the potential possibility to inhibit individual Oct-1 isoforms using the GSK3 inhibitor CHIR, an aminopyrimidine derivative that has a high affinity for GSK3α basic kinase and inactivates it. CHIR is an activator of the Wnt signaling pathway, and one of the best studied GSK3 targets is B-catenin, the key component of the Wnt signaling pathway. B-catenin is inactivated by active GSK3 kinase, which phosphorylates B-catenin, after which it enters the proteosome and degrades. Active non-phosphorylated B-catenin interacts with transcription factors of the TCF/LEF family and activates the transcription of genes of the Wnt signaling pathway. However, B-catenin can also participate in alternative transcription regulation pathways [12, 13].

Since the B-catenin protein is one of the most studied substrates for GSK3, we first studied the effect of CHIR on the expression of this protein in Namalwa cells. We used the primary antibodies capable of binding to only the non-phosphorylated (transcriptionally active) free B-catenin.

In this experiment, we showed that CHIR significantly increases the concentration of free B-catenin in the cytoplasm of Burkitt Namalwa lymphoma cells. Thus, it was found that CHIR is suitable for further work with the Namalwa cell line. In addition to the concentrations shown in Fig. 1, we also tested various concentrations of CHIR (0.6 μM, 0.4 μM, 0.1 μM, and 0.05 μM). The minimum concentration of CHIR sufficient for a visually detectable increase in the level of B-catenin in Namalwa cells was 0.05 μM.

**Fig. 1.**
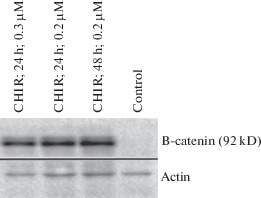
Changes in the amount of B-catenin in Namalwa cells during treatment with CHIR. Western blot hybridization with antibodies to non-phosphorylated B-catenin; 10 μg of total cellular protein was applied to the lane.

**CHIR suppresses transcription from the tissue-specific promoter of the** ***POU2F1*** **gene.**

At the next stage, we studied the effect of CHIR on transcription from two alternative promoters of the *POU2F1* gene by real-time PCR coupled to reverse transcription. PCR was performed using the following primers: Oct-1A A-Forw 5'-tattcaaaatggcggacgga-3'; Oct-1L L-Forw 5'-ccaccccaaactgctacctgt-3'; reverse primer for all isoforms was Rev-A,L 5'-ctgacggattgttcattcttgagt-3'. We showed that, when Namalwa cells were cultured for 24 h in a CHIR-containing medium, the amount of mRNA of the Oct-1L isoform, which is transcribed from the alternative tissue-specific promoter L, decreased approximately 1.8 times, whereas the amount of mRNA of the ubiquitous Oct-1A isoform did not change (Fig. 2). The decrease in the amount of Oct-1L mRNA depends on the CHIR concentration.

**Fig. 2.**
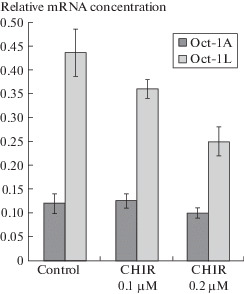
Effect of CHIR on transcription from alternative U and L promoters of the *POU2F1* (*Oct-1*) gene. Quantitative PCR.

The presented results indicate that CHIR suppresses transcription from the L promoter (from which the tissue-specific Oct-1L isoform is transcribed) but does not affect the transcription of the U promoter (from which the ubiquitous Oct-1A isoform is transcribed).

**CHIR causes a decrease in the amount of Oct-1L protein in Namalwa cells.**

Simultaneously, we analyzed the change in the amount of protein of the Oct-1L and Oct-1A isoforms by Western blot hybridization with antibodies specific to the Oct-1L isoform or to the Oct-1A isoform [4]. The results of the experiment showed that, under the influence of CHIR, the amount of Oct-1L isoform significantly decreased, whereas the amount of Oct-1A did not change (Fig. 3).

**Fig. 3.**
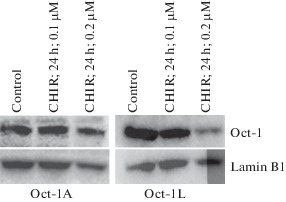
Effect of CHIR on the expression of Oct-A and Oct-L isoforms in Namalwa cells. Western blot hybridization with antibodies to Oct-1A or Oct-1L isoforms; 15 μg of total cellular protein was applied to the lane.

Thus, CHIR specifically affects the expression of the tissue-specific isoform Oct-1L, significantly reducing the level of mRNA and protein of the Oct-1L isoform; however, CHIR does not change the amount of mRNA and protein of the Oct-1A isoform. The analysis of binding sites showed that, in the regulatory region of the L promoter, there are several potential binding sites for the target proteins of GSK3—transcription factors of the TCF and FOXO families, whereas the U promoter contains the binding site for TCF7L2.

CHIR is considered an activator of the Wnt signaling pathway, because it highly specifically binds GSK3, thereby inactivating it and preventing phosphorylation of B-catenin by this kinase and its further degradation. It is known that the TCL-B–catenin complex on the promoter of target genes causes transcription activation. Both promoters of the *POU2F1* gene contain the binding sites for TCL. However, under the influence of CHIR, the repression of transcription from the tissue-specific promoter is observed, whereas the transcription from the ubiquitous promoter does not change. This suggests that GSK3 alters transcription from the alternative promoters of the *POU2F1* gene in a way other than the Wnt signaling pathway. To confirm this assumption, we used pyrvinium, a highly specific inhibitor of the Wnt signaling pathway. Treatment of Namalwa cells for 24 or 48 h with pyrvinium at a concentration of 2.2 μM did not cause a change in the level of transcription from the alternative promoters of the *POU2F1* gene. This fact confirms our assumption that the GSK3 inhibitor suppresses transcription from the tissue-specific promoter of the *POU2F1* gene not through the Wnt signaling pathway.

We assume that the GSK3 target is FOXO, the binding sites for which we found in the tissue-specific promoter L. It is known from the literature that GSK3 upregulates FOXO1 activity in humans and stimulates IGF-IR expression [14]. It was also shown that GSK3 directly activates dFOXO in *Drosophila melanogaster*.

Phosphorylation of proteins by GSK3 kinase can both positively and negatively affect the target protein function. Phosphorylation of MafA, SRC-3, and BCL-3 proteins induces either activation or degradation and changes their activity [9, 14, 15].

We have shown the effect of CHIR, a GSK3 inhibitor, on the decrease in the amount of mRNA and protein of the Oct-1L isoform in Namalwa tumor cells, and we assume that this influence is mediated by FOXO. We have previously shown that the expression level of this isoform in Burkitt Namalwa lymphoma cells is several times higher than in normal B cells. These results demonstrate that it is possible to develop a system for selective inhibition of Oct-1 transcription factor isoforms in human cells to suppress drug resistance of tumor cells with a high Oct-1 content.
